# Bacterial Quality and Molecular Detection of Food Poisoning Virulence Genes Isolated from Nasser Lake Fish, Aswan, Egypt

**DOI:** 10.1155/2024/6095430

**Published:** 2024-06-13

**Authors:** Nady Khairy Elbarbary, Mohamed K. Dandrawy, Ghada Hadad, Maha Abdelhaseib, Amna A. A. Osman, Rawaf Alenazy, Ibrahim Elbagory, Neveen M. Abdelmotilib, Fagelnour Elnoamany, Ghada A. Ibrahim, Reda A. Gomaa

**Affiliations:** ^1^ Department of Food Hygiene and Control Faculty of Veterinary Medicine Aswan University, Aswan 81528, Egypt; ^2^ Department of Food Hygiene and Control Faculty of Veterinary Medicine South Valley University, Qena 83522, Egypt; ^3^ Department of Animal Hygiene and Zoonoses Faculty of Veterinary Medicine University of Sadat City, Sadat, Egypt; ^4^ Department of Food Hygiene Safety and Technology Faculty of Veterinary Medicine Assiut University, Assiut 71526, Egypt; ^5^ Department of Food Science and Technology Faculty of Agriculture and Natural Resources Aswan University, Aswan 81528, Egypt; ^6^ Department of Medical Laboratory College of Applied Medical Sciences-Shaqra Shaqra University, Shaqra 11961, Saudi Arabia; ^7^ Department of Pharmaceutics Faculty of Pharmacy Northern Border University, Rafhaa 76321, Saudi Arabia; ^8^ Department of Food Technology Arid Lands Cultivation Research Institute (ALCRI) City of Scientific Research and Technological Applications (SRTA-CITY), New Borg El-Arab City 21934, Egypt; ^9^ General Administration for Laboratories Affairs National Food Safety Authority (NFSA), Cairo, Egypt; ^10^ Department of Bacteriology Agriculture Research Center (ARC) Animal Health Research Institute, Ismailia 41511, Egypt

## Abstract

The microbial analysis of fish is critical for ensuring overall health. Uncooked fish can serve as a conduit for transmitting several types of microbes; the current investigation sought to assess the bacterial levels in various kinds of fish from Nasser Lake, Aswan, Egypt, considered the chief source of potable water in Egypt. Two hundred and fifty fish samples, including 50 of each *Oreochromis niloticus*, *Sander lucioperca*, *Lates niloticus*, *Clarias gariepinus*, and *Mormyrus kannume*, from Nasser Lake, Aswan, Egypt, were collected to detect the bacterial load, isolation, and identification of *Aeromonas hydrophila*, *Pseudomonas aeruginosa*, and *Vibrio parahaemolyticus* and their virulence genes. The findings revealed that *Oreochromis niloticus* and *Clarias gariepinus* exhibited higher bacterial loads than other fish species. Incidences of bacterial contamination among examined fishes were 28.8%, 20.4%, and 16% for *Aeromonas hydrophila*, *Pseudomonas aeruginosa*, and *Vibrio parahaemolyticus*, respectively. Additionally, PCR analysis detected the presence of *aerA* (60%) and *Act* (40%) genes in *A. hydrophila*, *rpoB* (70%) and *LasB* (30%) genes in *P. aeruginosa*, and *ToxR* (70%) and *tdh* (50%) genes in *V. parahaemolyticus*. The study suggested that the bacterial contamination levels in *Oreochromis niloticus* and *Clarias gariepinus* could be notably more significant than in other species that could potentially be harmful to the consumers, especially considering the identification of particular bacteria known to cause foodborne illnesses. Further recommendations emphasized that regular monitoring and assessments are required to preserve their quality.

## 1. Introduction

Fish is an excellent source of many essential nutrients for human health, including high-quality protein, a plentiful supply of omega-3 fatty acids, vitamins, necessary amino acids, phosphates, and calcium [1]. Egypt currently generates over 73.8% of all fish raised in Africa and ranks ninth globally, producing 1.54% [2]. Despite the advantages, contamination is still possible due to bacteria or other physical, chemical, or biological contaminants. Bacterial contamination is the primary cause of fish food contamination among the dangers [3]. Microbial growth and residence in food are the primary factors contributing to food spoilage, producing undesirable metabolites that impart unpleasant flavours and aromas. This environment produces undesirable metabolites, which impart unpleasant flavours and aromas to the food [4]. Foodborne diseases are recognized to occur regularly and are related to low-income countries, probably due to improper food handling and hygiene, lack of food safety laws and weak implementation systems, lack of economic assets to procure safety tools, and lack of education and training for different food handlers [5]. Foodborne pathogens are those microorganisms which cause human diseases via virulence machinery, even sometimes at their low infectious dose [6]. Fish is contaminated with pathogenic and spoilage bacteria at any production and supply chain stage [7].

A variety of pathogenic bacterial species, including *E. coli*, *Salmonella* spp., *Staphylococcus aureus*, *Aeromonas* spp., *Pseudomonas* spp., and *Vibrio* spp., are responsible for fish foodborne illnesses [8]. *Salmonella* spp. are a significant public health concern since they are the most commonly documented cause of occasional cases and outbreaks of gastroenteritis globally [9]. Within the same framework, *E. coli* is a paramount bacterial contaminant, frequently employed as a surveillant for fish deterioration and contamination [10]. Moreover, among food poisonings, the highlights are those caused by *S. aureus*, which is responsible for food poisoning outbreaks worldwide [11]. Psychrotolerant *Pseudomonas aeruginosa* is the most commonly isolated bacterium from spoilt seafood; it causes unpleasant smells and odours in addition to slime, which degrades the product's quality. Many virulence factors, some of which are related to the bacterial cell surface and include lipopolysaccharide, flagella, type IV pili, type III secretion system, exotoxin A, proteases, and alginate, are linked to *P. aeruginosa*'s pathogenicity. These factors also aid in transforming active proteins and the adherence and colonisation of bacteria within a host cell [12].

Furthermore, *Aeromonas hydrophila*, widely distributed in aquatic environments, can not only cause diseases in fish and humans directly but also increase histamine levels, posing a chemical hazard to human health [13]. The pathogenicity of *A. hydrophila* has been related to numerous putative virulence agents, such as aerolysin (*aerA*) and cytotoxic enterotoxin (*Act*) [14]. In addition, *Vibrio parahaemolyticus* is the most researched *Vibrio* spp., and it is known to be harmful to people, causing gastroenteritis and diarrhoea in people worldwide [15]. Certain virulence factors, including tdh and *toxR*, are present in *V. parahaemolyticus* strains and are primarily associated with hemolysis and cytotoxicity in the host cell [16]. The microbiological contamination of fish and fishery products is associated with aquatic environments and the sanitary conditions from the farm to the table, including cultivation, harvest, processing, storage, and transportation. Thus, ensuring food safety is the responsibility of the authorities and those who produce and market the processing product [17].

Therefore, it was crucial to monitor the bacterial load and its pattern of occurrence in fish to offer relevant information on the risk profile of fish for public health. Furthermore, the traditional method could be more efficient and time-consuming, with only one test possible. Consequently, the conventional culture technique has proven inadequate in identifying the proliferating multitude of bacterial species. Polymerase chain reaction (PCR) is one of the most common molecular techniques, and it is widely used to detect fish pathogenic bacteria [5] rapidly. Nevertheless, despite numerous studies investigating various fish species sourced from aquacultures in Egypt, there needs to be more information concerning the safety and quality of freshwater fish from sources such as Nasser Lake, a critical source for Egypt's national fisheries. *Oreochromis niloticus* (Nile tilapia), *Lates niloticus* (Nile perch), *Sander lucioperca* (pike perch), *Clarias gariepinus* (African catfish), and *Mormyrus kannume* (elephant snout) are the most commonly captured in South Egypt's Nasser Lake [18] and one of the most extensively consumed fish species in Egypt [16, 19]. Therefore, this work assesses fish safety and bacterial quality at Nasser Lake, Egypt.

## 2. Material and Methods

### 2.1. The Research Site

**Figure 1:**
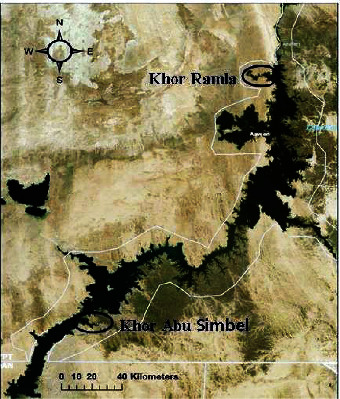
Map of Nasser Lake showing the position of Khor Ramla and Khor Abu Simbel.

The latitudes of Nasser Lake are 22°00′ to 23°58′N, while the longitudes are 31°19′ to 33°19′E [20]. It covers approximately 5248 km^2^ and has numerous side expansions (khors). The obtained fish were from two khors situated on the northern-western and southern-eastern shores of Nasser Lake (El-Ramla Khor and Khor Abu Simbel, respectively) during March and April 2023 (Figure 1).

### 2.2. Sample Collection and Preparation

Two hundred and fifty freshly caught fish samples (each weight ~200 g and ~30 cm length) include 50 of each *Oreochromis niloticus* (Nile Tilapia), *Sander lucioperca* (pike perch), *Lates niloticus* (Nile perch), *Clarias gariepinus* (African sharptooth catfish), and *Mormyrus kannume* (elephant snout) were collected from Nasser Lake in Aswan, Egypt, during March to April 2023. The samples were placed in sterile bags and refrigerated in an ice box before being sent to the laboratory. A sterile stainless-steel knife removes the scales, head, fins, tails, and bones after the fish has been eviscerated, and the two back fillets were kept in the refrigerator at 4°C for further examination. Aseptically, 25 g of flesh samples were put into stomacher bags with 225 ml of peptone water and then blended at 200 rpm for 2 min (Seward™ Stomacher™ Model 400 Circulator Lab Blender, 110 V). Homogenates were serially diluted in peptone water at 0.1% (nonselective preenrichment) at 37°C for 24 hr. One millimeter from each previously prepared serial dilution was inoculated separately into three appropriately marked triplicated Petri dishes for the following analysis:

1. *Total aerobic count (TAC):* ascertained by applying the pour plating technique with plate count agar (M091A, HiMedia) and incubating the plates for 48 hr at 35°C. The result is expressed as log cfu/g [21]
2. *E. coli count:* one millimeter of each serial dilution was spread-plated on Eosin Methylene Blue (EMB) agar (HiMedia MM022) using a conventional plate count method. Plates were incubated for 48 hr at 37°C. The number of metallic sheen colonies on plates ranging from 30 to 300 was determined utilising a digital colony counter (DC-8 OSK 100086, Kayagaki, Japan), and the result was expressed as log cfu/g [22]
3. *Staphylococcus aureus count:* one millimeter of each serial dilution was incubated at 35°C for 48 hr on Baird Parker Agar Base (Oxoid CM 0275) with egg yolk tellurite emulsion. After incubation, black, glossy colonies with translucent zones were identified and counted as *S. aureus*, and the finding was expressed as log cfu/g [23]
4. *Salmonella count:* 0.1 ml of nonselective preenrichment was placed into 10 ml of Rappaport-Vassiliadis broth (CM0866, Oxoid) and incubated at 42°C for 24 hr. A loop of selective enrichment broth was scattered onto Xylose Lysine Deoxycholate (XLD, CM0469, Oxoid) agar and incubated at 37°C for 24 hr using a digital colony counter. Suspected *Salmonella* colonies that showed up as red colour with black cores on XLD agar were counted and expressed as log cfu/g [24]
5. *Aeromonas hydrophila isolation and identification:* 1 ml of the homogenate was incubated for 24 hr at 28°C in 9 ml of brain heart infusion broth (BHI). Streaked *Aeromonas* isolation medium base agar was fortified with ampicillin (Biolife, CN0801) with one loop of enrichment broth, and the mixture was then aerobically incubated for 18 to 24 hr at 37°C. Green colonies with dark centers suggest *A. hydrophila* [25]. Preliminary screening and identification were conducted using gram staining, oxidase, and catalase tests. Gram-negative, oxidase, and catalase-positive isolates were preserved on a blood agar slant, and further biochemical characterizations such as hydrogen sulphide, indole test, urease test, Voges-Proskauer test, and sugars (glucose, inositol, and mannitol) were performed [26]
6. *Pseudomonas aeruginosa isolation and identification:* 1 ml of the previous dilution was dispersed with *Pseudomonas* agar base medium (M085, HiMedia) improved with glycerol, and the regionalized colonies (greenish-yellow colonies) were produced after 48 hr of incubation at 25°C [27]. The purified *P. aeruginosa* colonies were identified biochemically [28]
7. *Vibrio parahaemolyticus isolation and identification:*a loopful of nonselective preenrichment was streaked onto thiosulfate-citrate-bile salt-sucrose agar (GM189, HiMedia), and the mixture was incubated for 24 hr at 37°C. The presumed *V. parahaemolyticus* (smooth green colonies) was collected, purified, and biochemically identified [29]
8. *Determination of some food poisoning virulence genes:* 10 randomly obtained pure isolates of *A. hydrophila*, *P. aeruginosa*, and *V. parahaemolyticus* were incubated in nutrient broth for 24 hr at 37°C. Predefined *act* and *aerA* genes in *A. hydrophila*, *rpoB* and *LasB* genes in *P. aeruginosa*, and *tdh* and *ToxR genes* in *V. parahaemolyticus* were targeted using the sequences of the primers (Table 1) synthesised by Willowfort Company (United Kingdom) with the thermocycling program shown in Table 2, and according to its manufacture instructions, the extraction of genomic DNA from bacterial isolates using the GeneJET Genomic DNA Purification Kit (Catalog no. K0721, Thermo Scientific, USA) and *COSMO* PCRRED Master Mix (Code no. W1020300X, Willowfort, United Kingdom) was resolved on 1.5% agarose gel electrophoresis. Animal Health Research Institute, Dokki, Giza, Egypt, provided positive control for each target gene

### 2.3. Statistical Analysis

Performed statistical analysis to ascertain the prevalence of bacterial contamination in various fish species. Descriptive statistics were primarily used, with percentages calculated to represent the proportion of fish samples contaminated by each species of bacteria. ANOVA was evaluated to analyze all the findings statistically, and a *p* ≤ 0.5 was considered significant.

## 3. Results

Concerning the bacterial load in the examined samples, Table 3 reveals that the mean of the total aerobic count (log cfu/g) of the analyzed fish was the highest in *M. kannume* (5.73 ± 0.24) and *O. niloticus* (5.62 ± 0.39) followed by *C. gariepinus* (5.08 ± 0.36), while *L. niloticus* (4.32 ± 0.3) and *S. lucioperca* (4.23 ± 1 × 0.3) recorded the lowest value. Furthermore, all samples were accepted according to the Egyptian National Food Safety Authority (NFSA) [33] as TAC < 10^6^ cfu/g for fresh fish. Furthermore, the mean value of *E. coli* count (log cfu/g) was 4.48 ± 0.15, 4.65 ± 0.23, 5.85 ± 0.23, 6.11 ± 0.37, and 3.75 ± 0.14 for *O. niloticus*, *L. niloticus*, *S. lucioperca*, *C. gariepinus*, and *M. kannume*, respectively (Table 4). Furthermore, all samples did not match NFSA regulations, and there is a significant difference between the examined samples at *p* ≤ 0.05. On the other hand, Table 5 shows that the mean of the *S. aureus* count was 5.94 ± 0.42, 4.1 ± 0.23, 3.20 ± 0.32, 5.04 ± 0.39, and 3.03 ± 0.21 log cfu/g for *O. niloticus*, *L. niloticus*, *S. lucioperca*, *C. gariepinus*, and *M. kannume*. According to the NFSA, the accepted count of *S. aureus* was <10^4^. As a result, all tested samples were approved for *L. niloticus*, *S. lucioperca*, and *M. kannume*. At the same time, only 54% of *O. Niloticus* and 60% of *C. gariepinus* were accepted, according to the NFSA. Additionally, the findings shown in Table 6 show that the mean count of *Salmonella* spp., *C. gariepinus*, and *M. kannume* recorded the highest value with a mean of 6.42 ± 0.36 and 5.81 ± 0.22 log cfu/g, followed by *L. niloticus* and *O. niloticus* with a mean of 5.48 ± 0.39 and 5.32 ± 0.48 log cfu/g, while *S. lucioperca* reported a mean value of 5.08 ± 0.25 log cfu/g. The samples contaminated with *Salmonella* did not match the regulations established by the NFSA. The variances between the specimens were significant at *p* ≤ 0.05.

**Figure 2:**
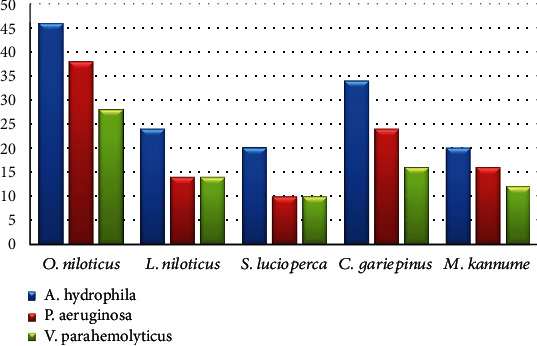
Occurrence of some bacterial contamination in the examined fish.

**Figure 3:**
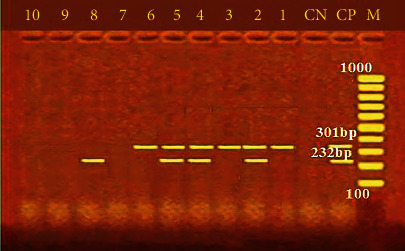
PCR electrophoresis of *A. hydrophila* virulence genes. M: 100 bp ladder; CP: positive control for *aerA* at 301 bp and *Act* at 232 bp; CN: negative control; lane (1, 2, 3, 4, 5, and 6): positive for *aerA* at 301 bp; lane (2, 4, 5, and 8): positive for *Act* at 232 bp.

**Figure 4:**
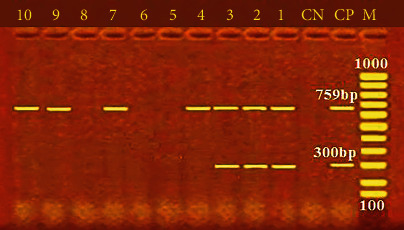
PCR electrophoresis of *P. aeruginosa* virulence genes. M: 100 bp ladder; CP: positive control for *rpoB* at 759 bp and *LasB* at 300 bp; CN: negative control; lane (1, 2, 3, 4, 7, 9, and 10): positive for the *rpoB* at 759 bp; lane (1, 2, and 3): positive for the *LasB* at 300 bp.

**Figure 5:**
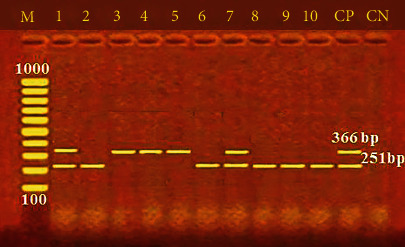
PCR electrophoresis of *V. parahaemolyticus* virulence genes. M: 100 bp ladder; CP: positive control for the *ToxR* at 366 bp and the *tdh* at 251 bp; CN: negative control; lane (1, 3, 4, 5, and 7): positive for the *ToxR* at 366 bp; lane (1, 2, 6, 7, 8, 9, and 10): positive for *tdh* at 251 bp.

Furthermore, Figure 2 shows the incidence of some bacterial contamination among the examined fish samples. 28.8% of the analyzed fish samples had *A. hydrophila* distributed as 46%, 24%, 20%, 34%, and 20% in *O. niloticus*, *L*. *niloticus*, *S. lucioperca*, *C. gariepinus*, and *M. kannume*, respectively. Moreover, 20.4% of the inspected fish had *P. aeruginosa*, with distribution rates of 38%, 14%, 10%, 24%, and 16% in O*. niloticus*, *L. niloticus*, *S. lucioperca*, *C. gariepinus*, and *M. kannume*, respectively. *V. parahaemolyticus* was recognized in 16% of examined fishes, with incidences of 28%, 14%, 10%, 16%, and 12% in *O. niloticus*, *L. niloticus*, *S. lucioperca*, *C. gariepinus*, and *M. kannume*, respectively. Differential biochemical tests confirmed that all positive isolates at the species level were *A. hydrophila* (Table 7). Moreover, the results of the occurrence of different virulence genes in the studied bacteria were confirmed by PCR.  Figure 3 shows the PCR electrophoresis of *A. hydrophila* virulence genes (*aerA* and *Act*), identified at 60% and 40%, respectively.  Figure 4 also displays the PCR electrophoresis of *P. aeruginosa* virulence genes, with incidence rates of the *rpoB* and *lasB* genes at 70% and 30%, respectively. Besides, 70% and 50% of the examined isolates had the *toxR* and *tdh* genes of *V. parahaemolyticus* (Figure 5).

## 4. Discussion

The microbiological status of fish meat is an essential marker of the sanitary conditions relevant to the environment for fish breeding, handling, and storing, as fish is a safe diet and healthy fish muscles are considered sterile [13]. The total bacterial count, which represents a significant guide for evaluating the level of meat cleanliness and hygienic value, plays an integral role in this process. Consequently, the information obtainable in Table 3 demonstrated the variations in the total aerobic count (log cfu/g) observed in the inspected fish. There were significant variations across the studied samples (*p* ≤ 0.05). TAC was the highest in *M. kannume* and *O. niloticus*, followed by *C. gariepinus*, while *L. niloticus* and *S. lucioperca* recorded the lowest value. According to NFSA [33], the allowable count of TBC was <10^6^; therefore, the analysis of all samples was concluded to be acceptable. The microbial quality of fish significantly impacts food safety. Fish is an exceptionally perishable food item that must be preserved and handled carefully. Three broad categories can be used to categorise pathogenic bacteria and spoilage linked to fish: native bacteria found in fish's natural commensal microbiota, exogenous enteric bacteria from nearby contaminated water, and bacterial contamination during processing, storage, or food preparation.

Microbial deterioration is considered a primary factor in the change in fish muscle's acceptability as a raw material for the food industry. Pathogenic and spoilage microorganisms can be introduced into fish and fish products throughout the production and supply chain [13]. The variance in the initial bacterial load of the examined fish samples can be attributed to the microbial load of the water in which they reside and the proliferation of bacteria that thrive under storing conditions [17]. Fishes are affected by the bacterial load and the bacterial species that are present in the ecosystem; also, Ali et al. [34] showed that fishes collected from polluted sites (drainage) recorded higher bacterial loads (in different fish organs) than fishes collected from nonpolluted sites. The results supported Amuneke et al. [35] and Khairy et al. [36]. On the other hand, the current research findings were higher than those provided by Mamdouh et al. [37], who reported 4.83 ± 0.43 log cfu/g in *O. niloticus* and lower than the finding of Ali [38], who reported TAC of 6.36 to 7.89 log cfu/g for fishes in Abu Simbel Khor.

Among the range of pathogenic bacterial species that cause fish foodborne diseases is *E. coli*. The pathogenic strains of *E. coli* cause diarrhoea by producing and releasing toxins and can also cause food spoilage in fish [39]. The findings (Table 4) indicate the mean count of *E. coli* (log cfu/g); a statistically significant association was found (*p* ≤ 0.05) between the various sample types and the occurrence of *E. coli*. Detecting *E. coli* in some fishes might represent postharvest cross-contamination [40]. The permissible *E. coli* count, as specified by the NFSA [33], is < 4 log cfu/g, which has been given as the quality criteria for this organism. As a result, none of the fish samples evaluated in the present investigation met the required standard, suggesting that improper handling and improper hygiene might lead to the contamination of ready-to-eat foods, eventually affecting the consumers' health. The occurrence of this bacterium in food is directly related to faecal contamination. This bacterium is the most abundant facultative anaerobe of the human intestinal microflora. Furthermore, *E. coli* is broadly present in the intestinal tracts of warm-blooded animals, and *E. coli* in ready-to-eat foods is undesirable because it suggests poor hygienic conditions that lead to contamination [39]. The results obtained in the present study were consistent with the values indicated by Yohans et al. [39], which ranged from 4.45 to 5.89 log cfu/g in the investigated samples. The *E. coli* load in raw fish samples was higher than that observed by Dhanapal et al. [41], who found 4.67 log cfu/g, but lower than that identified by Wendwesen et al. [42], who found 4.63 log cfu/g in Nile tilapia fillet samples. This potential disagreement might arise from the difference in the sample size used, the ecosystem of the study area, or the sampling methods.

*Staphylococcus aureus* is an opportunistic common pathogen associated with colonising humans' skin and musical surfaces. *S. aureus* may grow in food using temperature and time, producing heat-stable intoxication and cooking-resistant enterotoxins [17]. *S. aureus* is not a normal microbiota of fish; therefore, its presence in fish indicates cross-contamination originating from the harvest area or improper handling by fish handlers [43]. The mean of *S. aureus* count ranges from 5.94 ± 0.42 log cfu/g in *O. niloticus* to 3.03 ± 0.21 log cfu/g in *M. kannume* (Table 5). The variances between the specimens were significant at *p* ≤ 0.05. According to the NFSA [33], the accepted count of *S. aureus* was <10^4^. As a result, all tested samples were approved for *L. niloticus*, *S. lucioperca*, and *M. kannume*. At the same time, only 54% of *O. niloticus* and 60% of *C. gariepinus* were accepted, according to the NFSA [33]. This result shows that it is essential to implement effective regulations and good hygiene practices in the fish industry to ensure food hygiene. The outcomes matched those noted by Danba et al. [44] and Budiati et al. [45] but were lower than the findings of Mamdouh et al. [37]. *S. aureus* constitutes a substantial public health concern due to heat-stable staphylococcal enterotoxins (SEs) during its growth. Even if food is heated before consumption, the toxins can still cause illness even after the bacterial cells are destroyed by heat [46]. *S. aureus* is a product of contamination of the aquatic environment where they come from and poor production and handling conditions in hygiene [17]. The difference in *S. aureus* count and incidence rates found in this study among examined samples and other studies may result from differences in fish handlers' processing and hygienic practices.

*Salmonella* spp. is not part of the healthy fish microbiota, and its presence indicates faecal contamination either from polluted water or cross-contamination during the production chain (unhygienic fish handling, fish processing, or marketing) [47]. *Salmonella* spp. is the primary source of intestinal infections in animals and humans, and these bacteria are responsible for millions of illnesses globally. Nontyphoidal *Salmonella* spp. is mainly associated with foodborne diseases, making it a significant zoonotic agent [48]. *Salmonella* can be found in various sources, including soil, water, animal faeces, insects, equipment surfaces, and food processing plants [49]. The findings shown in Table 6 show the mean count of *Salmonella* spp. The observed distinctions among the samples were statistically significant at *p* ≤ 0.05. The samples contaminated with *Salmonella* did not comply with the regulatory thresholds established by the NFSA [33]. The recovery rate in the current study result was higher, and this could be due to contamination of a water source, the absence of hygienic practices, and strict follow-up of this sector by the concerned authorities. These findings are lower than those of Tanyag et al. [50]. Freshwater fish can act as passive carriers of *Salmonella*, excreting it without showing any clinical symptoms. The results agreed with Yohans et al. [39] and Kagambèga et al. [51]. The occurrence of *Salmonella* in this investigation is comparatively higher than that recorded in other studies [52–54]. The differences in prevalence rates can be attributed to sample size and sample types. *Salmonella* spp. are found in animal or human reservoirs, and their presence in raw fish in this study suggests poor hygienic practices during production, handling, processing, and marketing, which could be a result of direct or indirect faecal contamination, posing a risk to people consuming raw or undercooked contaminated fish.

*Aeromonas hydrophila* is regarded as one of the primary significant foodborne zoonotic pathogens. They are the natural inhabitants of different aquatic environments, such as freshwater, brackish, and marine water. It causes severe health effects in people, such as meningitis, septic arthritis, diarrhoea (traveller's diarrhoea), fulminating septicemia, and gastroenteritis [54]. In this study, 28.8% of the analyzed fish samples had *A. hydrophila*, *O. niloticus* reported the highest percentage (46%), while *S. lucioperca* and *M. kannume* had the lowest percentage (20%) (Figure 2). Differential biochemical tests confirmed that all isolates at the species level were *A. hydrophila* (Table 7). A statistically significant difference was found among fish samples and the occurrence of *A. hydrophila* (*p* < 0.05). Fish can be contaminated with pathogenic bacteria by polluted water or by handling, processing, and unhygienic storage conditions [55]. Almost identical to the outcome (43.8%) was recorded by Wamala et al. [56] and Kishk et al. [57]. Moreover, a high finding was reported by Yohans et al. [39], who recorded that 76.6% to 80% of examined samples had *A. hydrophila*. In addition, Morshdy et al. [54] and Dhanapala et al. [41] observed *A. hydrophila* with 14% and 9.3% from freshwater fish.

The pathogenicity of *A. hydrophila* has been related to numerous putative virulence agents, such as aerolysin (*aerA*) and cytotoxic enterotoxin (*Act*) [14]. On the other hand, *A. hydrophila* virulence-associated genes (*aerA* and *Act*) were found in 60% and 40% of the inspected *A. hydrophila* isolates (Figure 3). These results matched Emeish et al. [58] and Elbarbary et al. [59], who recorded 64.3% and 60% of the inspected *A. hydrophila* isolates. The results were lower than those of Ahangarzadeh et al. [60], who detected *aerA* and *act* genes in 51.61% and 74.19%, respectively. Morshdy et al. [54] noticed *aerA* in 75% of the examined *A. hydrophila* isolates. Previous research has demonstrated that hemolysin and aerolysin donate to *A. hydrophila* pathogenicity in fish and humans, which allows the bacteria to colonise, replicate, and damage the host tissues [61]. Determining virulence determinants is the key to identifying the pathogenic potentials due to these virulence determinants' multifunctional and multifactorial roles in *A. hydrophila* pathogenicity [62]. Previous data indicate that *A. hydrophila* has modest changes in incidence rates, and according to Hafez et al. [63], the different species, sample location and time, geographic region, postcapture contamination, fish species, types of water, handling, and manipulations during fish handling, storage, and transportation can all be factors in the changes of *Aeromonas* species incidence.

*Pseudomonas aeruginosa* is a rod-shaped, gram-negative bacterium with a high pathogenic potential. It is classified as a member of the *γ*-proteobacteria and is abundant in various environments, including water, plants, soil, and animals [38]. Psychotolerant *Pseudomonas* species are the most commonly isolated bacterium from spoilt seafood; they cause unpleasant smells, odours, and slime, which degrade the product's quality [64]. *P. aeruginosa* infection, encompassing digestive tract infections, is prevalent in underdeveloped countries at a rate of 17% and in Europe at 11.5%. Enterotoxigenic *P. aeruginosa*-contaminated fish induces diarrhoea, gastrointestinal disorders, and skin infections, particularly in immunocompromised patients [65]. In the current study, 51% of the inspected fish had *P. aeruginosa*, and its incidence ranged from 10% in *S. lucioperca* to 38% in O*. niloticus* (Figure 2). Differential biochemical assays verified the identity of each isolate as *P. aeruginosa* at the species level (Table 7). Additionally, a significant difference was found among fish samples and the incidence of *P. aeruginosa* (*p* < 0.05). These recordings corresponded with the data from Abd El-Maogoud et al. [66], who noted similar frequencies (47.3%). A previous study by Mamdouh et al. et al. [39] confirmed higher occurrences (65.0%) of *P. aeruginosa* in the examined samples, while Ali et al. [38], Elbarbary et al. [59], and Abd El-Maogoud et al. [66] recorded lower incidences of 13.8%, 5.0%, and 29%. *Pseudomonas* is a part of the usual fish microflora and can be opportunistic and developed into virulent and disseminated in distressed fish [67]. The variation in the results between different species may be due to the difference in hygiene measures applied during catching, handling, freezing, storage, and processing [68].

By producing a wide range of virulence factors, *P. aeruginosa* can adjust to the unfavourable environment of its hosts and enhance the likelihood of illness and infection [69]. Many virulence issues, some of which are related to the bacterial cell surface and comprise lipopolysaccharide, flagella, type IV pili, type III secretion system, exotoxin A, proteases, and alginate, are linked to *P. aeruginosa*'s pathogenicity. These factors also aid in transforming active proteins and the adherence and colonisation of bacteria within a host cell [38]. The incidence of virulence genes of *P. aeruginosa* mainly identified in this study was 70% and 30% for *rpoB* and *lasB*, respectively (Figure 4). This finding validates the variability of *P. aeruginosa* reported by numerous publications [64, 65] and emphasises the identification method's great discriminating power based on the *rpoB* gene. Additionally, it was shown that *P. aeruginosa* had unique virulence genes, such as *lasB*, and this could be justified by the fact that *P. aeruginosa* secretes elastase (LasB), a metalloproteinase involved in host colonisation and tissue damage [30]. The findings of this study revealed lower incidence rates of *rpoB* and *lasB* genes in *P. aeruginosa* isolates compared to the analysis shown by Benie et al. [64], who detected *rpoB* and *lasB* I with incidences of 91.1% and 89.2%, respectively. Meanwhile, the current results were higher than those reported by Elbarbary et al. [59] and Shahrokhi et al. [70], who confirmed that the *lasB* gene (50%) had been the primary detected virulence gene in inspected *P. aeruginosa* isolates. These results indicate that some virulence factors assist bacterial establishment and colonisation on the surface of the host, while others expedite the invasion of numerous tissues. *P. aeruginosa* can invade tissue. *P. aeruginosa* also produces toxins and enzymes that disrupt carnal barriers by disrupting cell membranes despite the host's immune system [30].

*Vibrio* spp., prevalent in numerous types of seafood, are microbial foodborne pathogens of water sources that increase human vulnerability to hazards to public health [71]. *V. parahaemolyticus* stands out for its prevalence among pathogens carried by food of marine origin [72] and is recognized in 16% of inspected fishes, with incidences ranging from 28% in *O. niloticus* to 10% in *S. lucioperca* (Figure 2). Differential biochemical analyses identified all isolates as *V. parahaemolyticus* at the species level (Table 7). A statistically significant difference was found between fish samples and the incidence of *V. parahaemolyticus* (*p* < 0.05), suggesting the possibility of transmission of *V. parahaemolyticus* through the consumption of fish. Similar outcomes obtained by Ahmed et al. [73] recorded 15.1%. High outcomes were achieved by Asran et al. [31] and Morshdy et al. [74], who identified *V. parahaemolyticus* in 50% and 42.3% of inspected fish; meanwhile, Elbarbary et al. [59], Suresh et al. [75], and Yen et al. [76] reported low percentages of 2.3%, 3.89%, and 7.5%. A statistically significant difference was found between fish samples and the incidence of *V. parahaemolyticus* (*p* < 0.05).

Cross-contamination, improper handling, a lack of cleanliness, and temperature fluctuations during keeping may explain the low variation in *Vibrio* isolation incidences [77]. PCR-based assays targeting the highly conserved *toxR* and *tdh* genes in *V. parahaemolyticus* have become a prevalent molecular procedure for discovering and identifying *V. parahaemolyticus* in seafood samples [78]. The *toxR* gene, linked to the cytotoxic activity and hemolysis of *V. parahaemolyticus* in the host cell, confirms the pathogenicity of *V. parahaemolyticus* connected with seafood [73]. In the current study, 70% and 50% of the examined bacteria had *toxR* and *tdh* genes, respectively (Figure 5). A high rate (100%) of *V. parahaemolyticus* isolates harboured the *toxR* gene by Morshdy et al. [74], Yen et al. [76], and Shahrokhi et al. [70], while Almejhim et al. [52] found a lower percentage (21.7%) held the *toxR* gene. The restricted number of strains and different primer designs may be to blame for the variations in the prevalence of virulence gene identification. It could also be explained by the species, various aquatic salinities, the quantity of fish investigated, and ecological conditions. Food safety is a worldwide health priority, and foodborne infections have a substantial global impact. Detecting microbiological pathogens in food is thus the solution to preventing and recognizing health and safety hazards.

## 5. Conclusion

The current study focused on examining fish available on the market and popular among consumers in Aswan, Egypt, concerning consumer safety. Additionally, the present study revealed that most fish samples had higher bacterial burdens than recommended, and increased bacterial contamination observed in *O. niloticus* and *C. gariepinus* compared to other species may constitute health hazards, mainly due to the detection of some food-poisoning bacteria such as *A. hydrophila*, *P. aeruginosa*, and *V. parahaemolyticus*. More research was needed to assess fish bacterial quality nationwide and develop faster, cheaper, and more sensitive methods for isolating and identifying foodborne pathogens and their virulence genes. Continuous enforcement of hygienic conditions in food handling, food contact surfaces, personal sanitary practices, and consuming fully cooked fish should be recommended to help eliminate foodborne pathogens.

## Figures and Tables

**Table 1:** The specific sequences of oligonucleotide primers for PCR.

Bacteria	Gene	Primer sequence (5′-3′)	bp	References
*P. aeruginosa*	*rpoB*	CAGTTCATGGACCAGAACAACCCG	759	[30]
		ACGCTGGTTGATGCAGGTGTTC		
	*LasB*	GGAATGAACGAG GCGTTCTC	300	
		GGTCCAGTAGTAGCGGTTGG		

*V. parahaemolyticus*	*tdh*	GTAAAGGTCTCTGACTTTTGGAC	251	[31]
		TGGAATAGAACCTTCATCTTCACC		
	*ToxR*	GTCTTCTGACGCAATCGTTG	366	
		ATACGAGTGGTTGCTGTCATG		

*A. hydrophila*	*aerA*	AACCGAACTCTCCAT	301	[32]
		TTGTCCGGGTTGTACTCGTC		
	*act*	GAGAAGGTGACCACCAAGAAC	232	
		AACTGACATCGGCCTTGAACTC		

**Table 2:** Thermocycling program for gene primers.

Bacteria	Target gene	Primary denaturation	Secondary denaturation	Annealing	Extension	No. of cycles	Final extension
*P. aeruginosa*	*rpoB*	94°C 3 min	94°C 60sec	58°C 60sec.	72°C 2 min	30	72°C 2 min
	*LasB*	94°C 5 min	94°C 60 sec	60 °C 1 min	72°C 1 min	35	72°C 7 min

*V. parahaemolyticus*	*Tdh*	94°C 5 min	94°C 1 min	55°C 1 min	72°C 1 min	30	72°C 7 min
	*ToxR*	94°C 10 min	94°C 1 min	63°C 1.5 min	72°C 1.5 min	20	72°C 10 min

*A. hydrophila*	*aerA*	94°C 5 min	94°C 30 sec	54°C 30 sec	72°C 1 min	30	72°C 10 min
	*Act*						

**Table 3:** Total aerobic count (log cfu/g) of analyzed fish (*n* = 50 each).

Sample	Minimum	Maximum	Mean ± SE	Accepted samples		
				NFSA^∗^	No.	%
*O. niloticus*	5.39	5.72	5.62 ± 0.39^a^	<10^6^ cfu/g	100	100
*L. niloticus*	4.83	5.61	4.32 ± 0.3^c^		100	100
*S. lucioperca*	3.62	5.70	4.23 ± 1 × 0.33^c^		100	100
*C. gariepinus*	3.67	5.26	5.08 ± 0.36^b^		100	100
*M. kannume*	4.92	6.08	5.73 ± 0.24^a^		100	100

^∗^Egyptian National Food Safety Authority [33] for fresh fish. *p* ≤ 0.04210 is considered a significant difference. The mean values with the same letters in each column do not show a significant difference.

**Table 4:** *E. coli* count (log cfu/g) among the analyzed samples (*n* = 50 each).

Sample	Minimum	Maximum	Mean ± SE	Accepted samples		
				NFSA^∗^	No.	%
*O. niloticus*	3.30	5.58	4.48 ± 0.15^c^	<4 × 10 cfu/g	0	0
*L. niloticus*	2.61	5.78	4.65 ± 0.23^c^		0	0
*S. lucioperca*	2.54	6.18	5.85 ± 0.23^b^		0	0
*C. gariepinus*	5.61	6.30	6.11 ± 0.37^a^		0	0
*M. kannume*	2.49	5.04	3.75 ± 0.14^d^		0	0

^∗^Egyptian National Food Safety Authority [33] for fresh fish. A difference at *p* ≤ 0.05 was regarded as significant. Same-letter mean values in each column do not significantly differ from one another.

**Table 5:** *S. aureus* count (log cfu/g) among examined samples (*n* = 50 each).

Sample	Minimum	Maximum	Mean ± SE	Accepted samples		
				NFSA^∗^	No.	%
*O. niloticus*	2.68	6.46	5.94 ± 0.42^a^	<10^4^ cfu/g	27	54
*L. niloticus*	2. 48	4.56	4.1 ± 0.23^b^		0	0
*S. lucioperca*	2.11	3.76	3.20 ± 0.32^c^		0	0
*C. gariepinus*	2.23	5.52	5.04 ± 0.39^a^		30	60
*M. kannume*	2.34	3.45	3.03 ± 0.21^c^		0	0

^∗^Egyptian National Food Safety Authority [33] for fresh fish. The investigated samples differ significantly at *p* ≤ 0.05. No discernible difference exists between values with identical letters in any column.

**Table 6:** *Salmonella* count (log cfu/g) among examined samples (*n* = 50 each).

Sample	Minimum	Maximum	Mean ± SE	Accepted samples		
				NFSA^∗^	No.	%
*O. niloticus*	4.98	5.48	5.32 ± 0.48^b^	Free	0	0
*L. niloticus*	5.20	5.58	5.48 ± 0.39^b^		0	0
*S. lucioperca*	5.70	6.41	5.08 ± 0.25^b^		0	0
*C. gariepinus*	5.56	6.30	6.42 ± 0.36^a^		0	0
*M. kannume*	5.52	6.00	5.81 ± 0.22^a^		0	0

^∗^Egyptian National Food Safety Authority [33] for fresh fish. Defined a significant difference at *p* ≤ 0.05. No discernible difference exists between values with identical letters in any column.

**Table 7:** Biochemical properties of isolated bacteria.

Characters	*A. hydrophila*	*P. aeruginosa*	*V. parahaemolyticus*
Gram stain	Negative	Negative	Negative
Shape	Rods	Rods	Rods
Motility	Motile	Motile	Motile
Catalase	+	+	+
Oxidase	+	+	+
H_2_S	+	−	−
Glucose fermentation	+	−	+
Growth in vibriostatic agent 0/129 at 10 *μ*g	+	−	−
Indole	+	−	+
Urease	+	−	+
Voges-Proskauer	+	−	−

## Data Availability

The article contains the requisite data to substantiate the conclusions drawn in this study.
